# Toward Human-Centered Artificial Intelligence for Users’ Digital Well-Being: Systematic Review, Synthesis, and Future Directions

**DOI:** 10.2196/69533

**Published:** 2025-09-10

**Authors:** Youngsoo Shin

**Affiliations:** 1 Seidenberg School of Computer Science and Information Systems Pace University New York City, NY United States

**Keywords:** digital well-being, digital health, human-centered AI, human-AI interaction, user experience, systematic literature review, artificial intelligence, mobile phone

## Abstract

**Background:**

As information and communication technologies and artificial intelligence (AI) become deeply integrated into daily life, the focus on users’ digital well-being has grown across academic and industrial fields. However, fragmented perspectives and approaches to digital well-being in AI-powered systems hinder a holistic understanding, leaving researchers and practitioners struggling to design truly human-centered AI systems.

**Objective:**

This paper aims to address the fragmentation by synthesizing diverse perspectives and approaches to digital well-being through a systematic literature review. Using the stimulus-organism-response framework as a guiding lens, this study aims to develop a comprehensive model for designing human-centered AI systems that promote digital well-being.

**Methods:**

A systematic review of 240 multidisciplinary publications was conducted to explore the intersection of AI, digital well-being, and human-centered design. The analysis involved identifying key themes, frameworks, and approaches, with the stimulus-organism-response model serving as an overarching perspective to organize findings and inform the model development.

**Results:**

The review led to the development of a human-centered artificial intelligence model for digital well-being, a conceptual framework that consolidates current knowledge on designing AI systems to support digital well-being and positively influence human behavior. The proposed model integrates insights from cross-disciplinary research, providing a structured understanding of how AI system features (stimuli) affect users’ internal states such as perceptions and emotions (organisms) and lead to attitudinal or behavioral changes (responses). Additionally, this paper highlights emerging challenges and opportunities, including ethical considerations, scalability, and practical guidelines for applying the model in long-term research and practice.

**Conclusions:**

This study contributes to advancing the field by presenting an overarching framework for fostering digital well-being through human-centered AI systems. By addressing gaps in the fragmented literature and proposing a unifying model, the findings offer insights for researchers and practitioners. The human-centered artificial intelligence for digital well-being model serves as a foundation for future exploration and practical application in creating intelligent computing systems that improve users' digital well-being in everyday life.

## Introduction

Digital well-being is a concept that describes the impact of information and communication technologies (ICTs) on people’s physical and mental well-being in general [1-3]. Researchers and practitioners in many relevant fields, including computer science, health care, human-computer interaction (HCI), psychology, cognitive science, and data science, have emphasized that there are plenty of opportunities to leverage everyday ICTs in users’ daily HCIs [4-14]. In this regard, daily intelligent computing systems have been developed and investigated mainly with a focus on people’s higher level of involvement in technology used to enhance their well-being and behavioral design [2,15].

Existing literature has provided diverse methods and approaches for building ICT-based computing systems that support users’ daily quality of life by promoting users’ positive attitudinal and behavioral changes [16]. For the past decade, the trend of increasing computing systems’ complexity and interactivity has stimulated the introduction of a variety of artificial intelligence (AI) technologies (eg, chatbots, voice assistants, self-driving vehicles, or recommendation systems) that have penetrated far into people’s everyday lives [17,18]. The application areas of these intelligent systems include (1) consumer applications such as recommender systems and social media platforms, (2) consequential applications in medical, legal, environmental, or financial systems, and (3) life-critical applications such as those in cars, airplanes, or military systems [19]. Furthermore, the previous COVID-19 pandemic has accelerated the use and adoption of these AI-powered intelligent systems in people’s lifestyles [20]. For example, the social distancing restrictions in everyday life during the pandemic have increased the demand for AI-powered services and noncontact operations such as driverless delivery services, pre-entry wellness checking systems, or autonomous cleaning solutions [21,22].

However, several studies have pointed out negative aspects of prior approaches and perspectives that maximize users’ engagement with these computing systems by attracting their attention and encouraging them to interact with the systems more frequently [1,23-26]. In this regard, large global technology companies, such as Google, Apple, Microsoft, and Meta, have reframed the meaning of digital well-being and have incorporated digital well-being into their business models. They have introduced various design and system features to help users manage their interactions with these computing systems and address these users’ challenges in terms of being overloaded and distracted by the systems [27]. Many of these features have been developed as personal informatics tools (PITs) or digital self-control tools. With an emphasis on an instant and short-term well-being–enhancing intervention, these tools have mainly allowed users to monitor and reflect on their technology use with digital timers, usage dashboards, or lock-out features [16,25].

The concept of digital well-being has been interpreted in a broad sense among relevant fields with a strong consideration of daily HCI and recent human-AI interaction [28]. However, as aforementioned, several researchers and practitioners have also construed in a limited sense of the term by sharing a consensus that “excessive” and “frequent” use of systems could result in physical or mental health problems for users [11,29-34]. Despite this perspective gap, there is still a lack of studies that systematically reframe the concept of digital well-being with AI-powered computing systems. Further, the varying approaches developed in the related disciplines tend to focus on domain-, device-, or context-specific and fragmented aspects of human experience and interactions with computing systems, which can be limiting for researchers and practitioners as they search for a holistic understanding of digital well-being in daily HCIs and human-AI interactions [2,15,25,35,36].

In response, this paper sees the value in consolidating diverse but scattered initiatives regarding designing human-centered AI systems for users’ digital well-being to support effective implementation by researchers and practitioners. More specifically, this paper addresses the research question as follows: “How can existing research on digital well-being with intelligent computing systems be reframed and synthesized toward an overarching model for human-centered AI?”

As part of the effort to develop such a cumulative body of knowledge, this paper conducts a multidisciplinary systematic literature review on intelligent computing systems for users’ daily digital well-being with a consideration of user- and human-centered perspectives. The findings from the review serve as a conceptual foundation for the development of an overarching model: human-centered artificial intelligence for digital well-being (HCAI-DW) model. The results from this consolidation are also expected to enrich the current understanding of digital well-being in daily HCIs for further implementation of designing human-centered computing systems.

## Methods

### Theoretical Framework: The Stimulus-Organism-Response Model

To address the current knowledge gaps, this study developed an initial conceptual model of human-centered computing for digital well-being. In this process, this paper incorporated the stimulus-organism-response (SOR) model. According to the SOR model [37,38], an individual’s responses to a stimulus are the result of a behavioral decision-making process that reflects interplay between internal aspects (eg, preference, personality, ability, motivation, etc) and external aspects of the specific context (eg, time, money, weather, etc). The SOR model states that either context or environment could be understood as a stimulus (S), which consists of a set of designed or nondesigned interactions that causes an internal cognitive process in individuals (O) and produces a response (R) [39,40].

The SOR model has allowed researchers and practitioners in various fields to investigate how humans interact with nonhuman objects broadly and comprehensively [39]. In this regard, many have incorporated the model because of its broader range of possibilities for human behavior and objects and its structured way of focusing on behavioral mechanisms instead of behavioral consequences (eg, satisfaction, performance, outcome, utility, or increased well-being status). For this reason, this paper seeks to bridge the existing perspective gaps by using the basic SOR model, with complementary points of view for digital well-being and human-centered AI computing systems.

**Figure 1:**
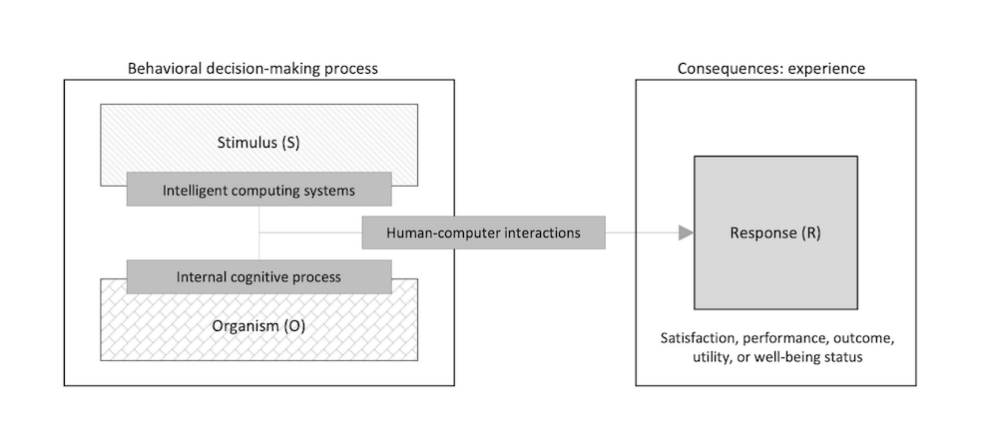
Overarching viewpoint of digital well-being with intelligent computing systems.

Through incorporating the SOR model as an initial viewpoint, it is expected that this study can investigate multilayered aspects of digital well-being with intelligent computing systems (Figure 1). Most previous perspectives on digital well-being in relevant fields mainly paid attention to the “consequences” of experiences, instead of focusing on the different ways people make behavioral decisions and their divergent experiential processes with intelligent systems and technologies. Researchers and practitioners should consider behavioral decision-making mechanisms as well as a specific behavioral result targeted by designed interactions, including interface and system features. However, a systematic understanding of the multidimensional aspects of the behavioral decision-making process within the research boundary of design for digital well-being with intelligent computing systems is still lacking. Therefore, from the perspective of the SOR model, this study concentrates on individuals’ behavioral decision-making mechanisms as a core concept for the unified conceptualization of digital well-being with intelligent computing systems.

### Data Collection

#### Overview

This study uses a systematic literature review to gain a structured understanding of diverse perspectives on digital well-being within intelligent computing systems. Systematic literature reviews are recognized as rigorous and transparent methods for synthesizing existing evidence and addressing emerging interdisciplinary issues [41]. This review was guided by 2 complementary methodological frameworks: the PRISMA (Preferred Reporting Items for Systematic Reviews and Meta-Analyses) guidelines [42] (Multimedia Appendix 1) and the approach developed by Tranfield et al (2003) [43], which is widely adopted in evidence-informed management research. Following these frameworks, the review was conducted in four phases: (1) searching existing research, (2) screening relevant studies and approaches, (3) reviewing and evaluating focal studies on digital well-being with intelligent computing systems, and (4) conducting supplementary backward and forward searching.

In the initial phase, 4 primary databases—Web of Science, EBSCO, PubMed, and the Association for Computing Machinery (ACM) Digital Library—were used to identify relevant studies. The first publication search was conducted on August 15, 2022, with the final literature list updated on May 20, 2025, to ensure the inclusion of up-to-date studies.

The search strategy was designed by analyzing prior research and examining terminology used in the relevant scientific papers, which were critically reviewed in this paper’s initial scoping review phase. For the searching process, the initial keyword string was designed to focus on the intersection of technology and well-being across multidisciplinary fields by constructing a query that combined multiple thematic dimensions:

- Core concepts: the query incorporated terms related to well-being, such as “wellbeing” and “well-being,” to capture diverse terminology used across disciplines.
- Technological scope: keywords such as “system,” “interface,” “computer,” and “technology” were included to ensure relevance to studies that involve digital and computational systems.
- Intelligence and interactivity: to refine the focus on intelligent and adaptive technologies, terms such as “artificial intelligence,” “AI,” “intelligent,” “interactive,” “smart,” “autonomous,” and “digital” were included.

#### Data Collection From the Web of Science

The Web of Science was chosen as the primary database for conducting a systematic literature search due to its comprehensive coverage of high-impact journals, conference proceedings, and other scholarly publications. The final query was adapted as follows:

TS=(“wellbeing” OR “well-being”) AND TS=(“system” OR “interface” OR “computer” OR “technology”) AND TS=(“artificial intelligence” OR “AI” OR “intelligent” OR “interactive” OR “smart” OR “autonomous” OR “digital”)

Boolean operators and wildcards were applied to broaden the search and accommodate variations in terminology. Advanced search filters were also used to focus on peer-reviewed publications, including journal papers, conference proceedings, and review papers. The systematic search yielded an initial pool of 5424 papers.

#### Data Collection From the EBSCO

The EBSCO database was used to conduct a comprehensive literature search, leveraging its extensive collection of resources spanning a wide array of academic disciplines. EBSCO was chosen for its strong interdisciplinary coverage, particularly in areas related to social sciences, computer science, and HCI, making it well-suited for exploring the digital aspects of well-being. The search string mirrored that of other databases:

(“wellbeing” OR “well-being”) AND (“system” OR “interface” OR “computer” OR “technology”) AND (“artificial intelligence” OR “AI” OR “intelligent” OR “interactive” OR “smart” OR “autonomous” OR “digital”)

The search was limited to peer-reviewed publications to ensure that only high-quality scholarly work was included. Additionally, in EBSCO, the search was restricted to the abstracts of papers to focus on studies that explicitly address the targeted themes. The initial query yielded a total of 6433 papers, encompassing a wide range of studies related to the HCI and digital well-being domains.

#### Data Collection From the PubMed

The PubMed database was chosen as a key resource for exploring studies at the intersection of digital well-being and intelligent systems due to its focus on digital health, biomedical research, and related fields. To align with the overarching research goals, the following search query was constructed to capture a wide range of relevant studies:

((“wellbeing” OR “well-being”) AND (“system” OR “interface” OR “computer” OR “technology”) AND (“artificial intelligence“ OR ”AI“ OR ”intelligent“ OR ”interactive“ OR ”smart“ OR ”autonomous“ OR ”digital“))

The initial query yielded 4073 papers. This output reflects the strength of PubMed in identifying multidisciplinary research that links intelligent systems with digital well-being, particularly within the contexts of health care and user interaction.

#### Data Collection From the ACM Digital Library

Finally, the ACM Digital Library was selected for its specialized focus on computing and information technology research, encompassing both theoretical advancements and practical insights from academia and industry. Given its prominence in the field of computing, the ACM Digital Library was anticipated to offer unique and highly relevant contributions to understanding the intersection of digital well-being and intelligent systems. The search was designed to explore themes of digital well-being, particularly in the context of HCI. To capture a broad range of studies, the following search query was used:

AllField:(”wellbeing“ OR ”well-being“ OR ”digital wellbeing“ OR ”digital well-being“)

To ensure high relevance and quality, 1 advanced filter was applied to limit the results to research papers published under the ACM SIGCHI (Special Interest Group on Computer-Human Interaction). SIGCHI represents a core community in HCI research, making it a critical source for studies at the nexus of well-being and intelligent systems. The initial search yielded a total of 5669 papers. This comprehensive set of results highlights the breadth of research contributions in the ACM Digital Library, particularly within the SIGCHI community, which has long been at the forefront of exploring the relationship between human-centered design (HCD), interaction, and user well-being.

### Final Literature Sample and Data Exploration

From the initial search, a total of 21,599 papers were identified. These papers were manually screened by the researcher based on their titles, abstracts, and index keywords, resulting in a reduced set of 218 papers. The manual screening process was guided by 5 predefined inclusion criteria (Textbox 1), which ensured consistency and relevance in the selection process.

**The eligibility criteria for selecting the papers for the final review process.**

- The paper should be related to the targeted research areas and topics of this study.
- The paper should be peer-reviewed and categorized as an academic paper.
- The paper should have well-defined research purposes to tackle the issue of design for digital well-being with intelligent computing systems in everyday contexts.
- The paper should point out the importance of investigating interactions between users and intelligent computing systems in their everyday behavioral decision-making contexts.
- The paper should contain practical aspects to create or evaluate the designed interactions for supporting users’ digital well-being.

Subsequently, the remaining papers were reviewed in full. During this phase, backward and forward citation searches were conducted, yielding 12 additional papers. To further enhance the comprehensiveness and objectivity of the review, 10 relevant papers were also identified through consultations with external researchers and practitioners with expertise in HCI. These external consultations provided valuable third-party perspectives and helped mitigate potential bias in paper selection. After the review process, a total of 240 papers were selected for the final literature sample.

After the data collection and screening process, an inductive qualitative coding approach was conducted to systematically analyze the full text of each selected publication. The coding process was guided by the SOR model, which served as a conceptual lens to identify and organize how each paper addressed the roles of stimulus (ie, technology or system-level factors), organism (ie, user-level individual and contextual factors), and response (ie, behavioral outcomes or digital well-being effects). A coding scheme was iteratively developed and refined during this process to capture the range of constructs and considerations described in the literature. Particularly, each paper was coded based on its entire content, allowing for the extraction of both explicit concepts and latent themes. The codes derived from this process were then used in an affinity diagramming procedure, wherein related codes were grouped and clustered to surface patterns across the corpus. This bottom-up synthesis approach enabled the researcher to identify conceptual similarities, contradictions, and gaps across the diverse literature on AI systems and digital well-being.

Through the affinity diagramming process [44], codes organically clustered into 3 major thematic areas. The first cluster focused on implementation domains of intelligent computing systems, capturing how technologies were designed to support overall well-being, facilitate behavior or habit change, or serve as behavioral intervention tools. The second cluster highlighted conceptualizations of human autonomy, including autonomy as supported through technology and autonomy from human control. The third cluster reflected each paper’s approaches to system design and evaluation, with codes centering on the need to incorporate contextual adaptation (stimulus), individual differences (organism), and outcome-specific tailoring (response) into the development and assessment of AI-driven digital well-being interventions.

These 3 clusters, developed through iterative coding and visual grouping via affinity diagramming, provided the conceptual foundation for a model of human-centered AI computing systems for digital well-being presented in this study. All selected papers and their coding outcomes are described in the attached Multimedia Appendix 2, which is a review of 240 publications from 2008 to 2024.

## Results

### Sample Data Description

**Figure 2:**
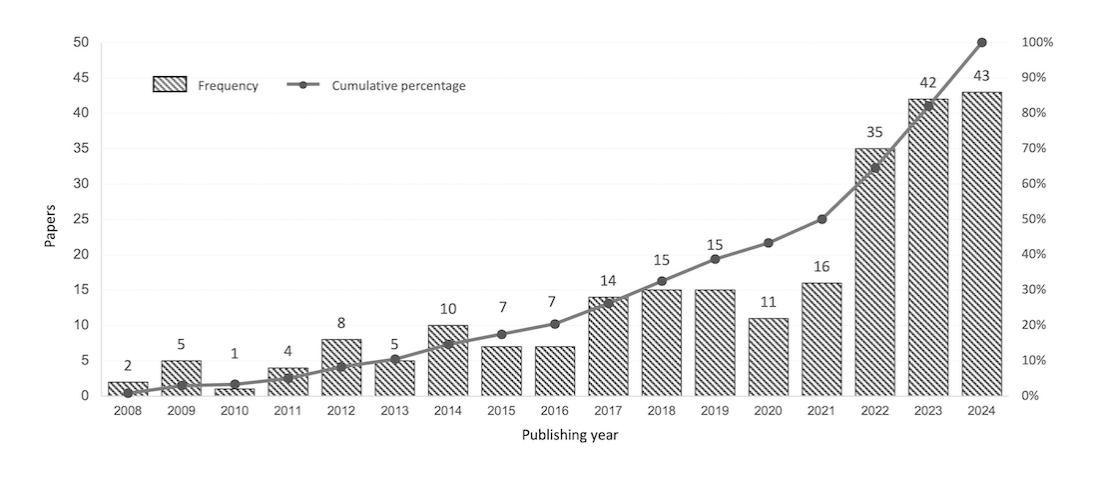
Publication years of the selected papers on digital well-being and intelligent computing systems.

**Figure 3:**
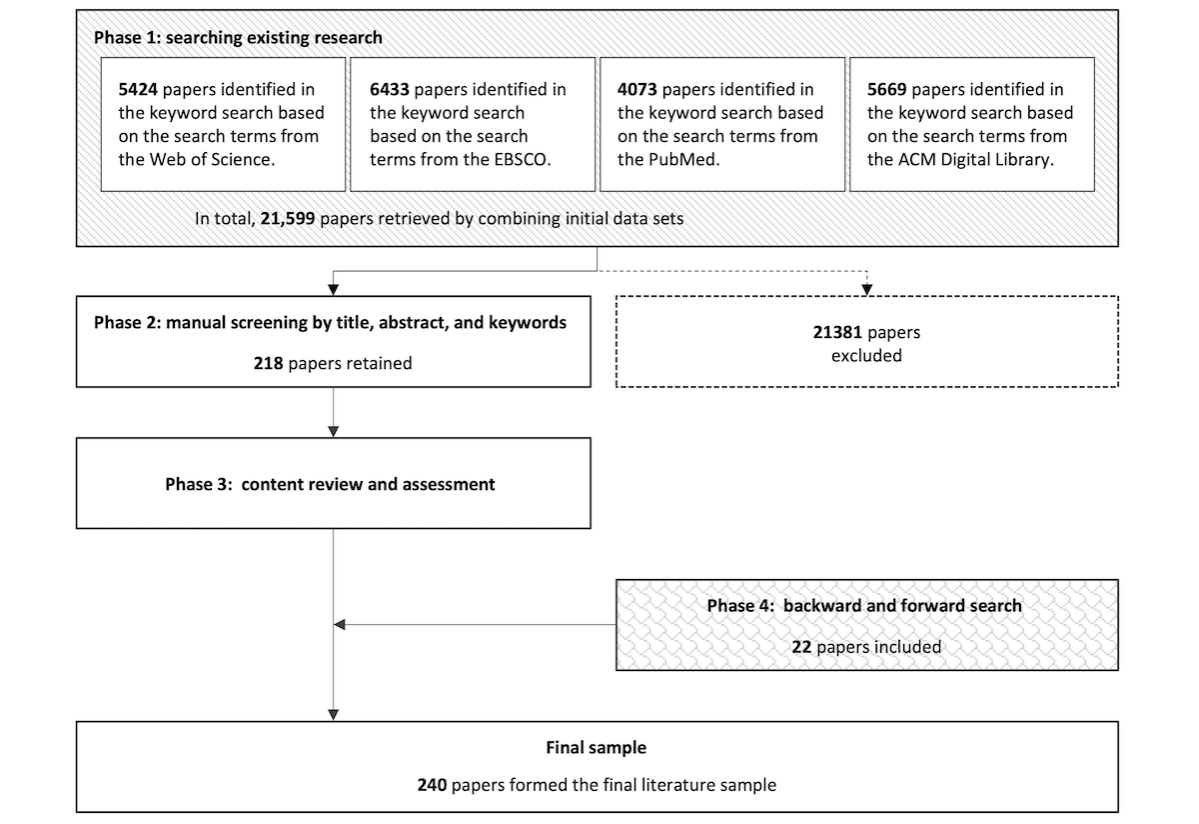
Overview of the data collection process. Databases: Web of Science, EBSCO, PubMed, and the Association for Computing Machinery (ACM) Digital Library.

The review of 240 publications between 2008 and 2024 reveals an evolving landscape in which digital well-being and intelligent computing systems have become central themes of inquiry across multiple disciplines (Figure 2). The temporal distribution of studies demonstrates a marked increase in scholarly attention, with the number of relevant publications growing from just 2 papers in 2008 to 43 in 2024. This acceleration reflects the growing urgency—both academic and societal—to understand how intelligent systems can be designed and evaluated to support human well-being in digital contexts. Figure 3 illustrates the data collection process.

During the initial period from 2008 to 2016, research primarily focused on early digital interfaces and the psychological impact of technologies such as smartphones, social media, and ubiquitous computing platforms. These studies were largely rooted in exploratory research, with conceptual frameworks drawn from subjective well-being and affective computing. The dominant focus was on describing emotional and behavioral responses to digital engagement, often framed in terms of stress, distraction, or mood regulation. The technological systems under study were mostly nonadaptive and nonintelligent, and user autonomy was often treated as a passive construct.

An inflection point emerged in 2017. This period marked a transition from exploratory inquiries into more normative and design-oriented investigations. Triggered by public discourse around technology addiction, ethical technology, and digital detox movements, scholars began reframing digital well-being as a design goal rather than merely a behavioral outcome. This era coincided with a surge in industry initiatives (eg, Apple’s Screen Time and Google’s Digital Wellbeing suite), and research began to reflect growing societal concern about how technologies could undermine or support flourishing. Tools such as digital self-control tools and PITs became common subjects of investigation, signaling a broader shift toward proactive design strategies for well-being.

From 2022 onward, there was a notable rise in publications proposing frameworks, systems, and user studies that examined the role of human-centered AI in supporting digital well-being. Interdisciplinary contributions expanded, including papers from HCI, health informatics, behavioral science, and even management science. Concepts such as autonomy, persuasive design, ethical nudging, and emotion-aware interfaces were increasingly investigated through user-centered lenses. Many of these studies moved from simple heuristics to formal models, measurement tools, and empirical evaluations of intervention efficacy.

By 2024, the scope of the literature had broadened further to include the intersection of digital well-being with AI. This more recent body of work introduced sophisticated technologies, including generative AI, adaptive learning systems, hybrid human-AI collaboration platforms, and emotion recognition interfaces. These systems increasingly relied on real-time user data to personalize feedback and optimize interventions.

Overall, the sample data reinforces the need for design frameworks that synthesize empirical insights, respect user autonomy, and adapt to contextual and personal differences. As explored in the following findings and the model development section, the coding analysis helped identify key pain points and opportunities across system implementation, user experience (UX), and evaluation design.

### Emerging Perspectives on Digital Well-Being and Intelligent Systems

#### Overview

From the in-depth analysis with the affinity diagramming process, 3 emerging perspectives were clustered, which can be incorporated into the development of a model of human-centered AI computing systems for digital well-being.

#### Finding 1. Three Implementation Areas of Intelligent Computing Systems for Digital Well-Being

As summarized in Table 1, the dataset revealed 3 major implementation areas—daily well-being in HCIs, design for behavioral change, and behavioral intervention technologies (BITs)—that form the basis for findings 1-3 in this study.

**Table 1:** Summary of 3 implementation areas in intelligent computing systems for digital well-being.

Implementation area	Description	Papers, n	Common theories or approaches	Key limitations
Daily well-being in HCIs^a^	Broad framing of well-being as a design goal, often informed by psychological theories	114	Self-determination theory and hedonic experience	Conceptual vagueness and limited operationalization
Design for behavioral change	Focus on changing user behaviors or habits via persuasive or adaptive systems	71	Dual-process theory, habit theory, and goal-setting theory	Lack of long-term efficacy and inconsistent success metrics
Behavioral intervention technology	Systematic approaches rooted in clinical settings using adaptive, data-driven interventions	55	BITs^b^ and personal informatics	Limited practical integration and focus on high-income populations

^a^HCI: human-computer interaction.

^b^BIT: behavioral intervention technology.

#### Daily Well-Being in HCI

First, well-being in daily interactions with computing systems has been studied through a variety of lenses (ie, digital health, technology for mental health, or digital medicine) and under diverse human behavioral contexts over the past 2 decades. Among these various perspectives, “well-being in HCIs” is the broadest for considering technology as a tool for human flourishing and well-being [3]. Most related works aim to improve a user’s psychological and subjective well-being by developing interactive design and technology interventions along 2 pathways. The first pathway involves formulating system design principles by using relevant theories from psychology, such as self-determination theory and hedonic experience [3,45,46]. The second pathway involves investigating certain well-being determinants derived from these theories—such as autonomy, competence, and relatedness—which typically serve as both intentions for computing system design and as criteria for evaluations in technology development processes [3].

The review of 240 papers confirmed that this conceptualization is foundational: 114 (48%) studies in the dataset treat well-being as a core design or evaluative objective. Many of these adopt an HCI-centric viewpoint, emphasizing positive emotional experiences, mental health, or life satisfaction as outcomes of well-designed user interactions. However, despite shared theoretical anchors—especially self-determination theory—there remains significant inconsistency in how well-being is defined, operationalized, and measured across studies. Most of the papers in this category relied on self-report measures (eg, user satisfaction surveys and qualitative feedback) to evaluate well-being outcomes. These findings reveal a methodological gap between theoretical aspiration and empirical implementation.

Previous studies have attempted to suggest tools for system design and development practices by grounding them in rigorous and evidence-based empirical research. However, the major criticism of the well-being perspective in HCIs has been the fragmented and unstructured nature of its implementation [3,47]. Furthermore, the notion of well-being is often used as a descriptive umbrella for current lifestyle trends or invoked conceptually to justify a human-centric design ethos, especially in discussions focused on the outcomes of system design [32]. As a result, many studies frame well-being as an aspirational goal but lack the definitional clarity or structured methodology required for robust evaluation.

Still, the literature reflects an evolution in the treatment of well-being. In earlier studies—particularly those published before 2016—the dominant approach was reactive: minimizing digital harms (eg, mitigating distraction, reducing screen time, or addressing smartphone addiction). More recent contributions, however, embrace a proactive stance. Instead of merely limiting technology’s negative impacts, intelligent systems are now increasingly designed to scaffold reflection, self-regulation, emotional resilience, and long-term behavior change.

Despite these promising developments, the field still lacks integrative models that bridge disciplinary boundaries and connect technological features to measurable well-being determinants. Moreover, the challenge of balancing persuasive design with respect for user autonomy remains unresolved, as detailed in finding 2. While the well-being domain continues to expand in scope and influence, future research must prioritize the development of validated, multidimensional frameworks that align well-being theory with empirical evidence and real-world system design practices.

#### Design for Behavioral Changes

Second, the topic of design for behavioral change has been explored from a more defined and targeted angle by focusing on both short-term and long-term changes in users’ behaviors [48]. In the context of digital well-being, designing for behavior change often involves addressing complex, subjective aspects of human experiences—elements that are inherently difficult to operationalize and quantify [49]. To address these challenges, researchers and practitioners have drawn extensively from behavioral science, using empirically validated frameworks that explain how human attitudes, cognitive patterns, and routines can be shaped through deliberate system design [28]. These behavioral foundations have been used to develop intelligent systems that aim to promote health-related behaviors and improve users’ overall well-being.

The systematic review of 240 papers identified 71 (30%) studies that explicitly frame their system design objectives around catalyzing behavioral or habitual changes through intelligent computing. These studies span a wide range of behavioral domains, including digital overuse, emotional regulation, sleep hygiene, and physical activity. What distinguishes this subset is its methodological rigor and theoretical alignment. Most of these studies ground their design logic in well-established behavioral science models, such as dual-process theory, habit formation theory, and implementation intentions. These theories provide a robust framework for creating interventions that influence users’ decision-making processes across varying contexts and levels of awareness.

More recently, research and practice in this domain have shifted toward exploring the long-term consequences of technology-mediated behavior and its cumulative impact on well-being. For example, several studies [50,51] have sought to move beyond optimizing discrete moments of interaction (eg, interface usability and prompt timing) to designing systems that encourage enduring lifestyle changes. As a result, the field is beginning to transition from a narrow focus on behavior change to a more expansive framing around habit change. The review indicates a growing number of studies—particularly those integrating machine learning models—that aim to detect recurring behavior patterns and trigger interventions based on behavior-specific cues [52]. These approaches reflect a deeper ambition: to influence not only immediate choices but also the broader cognitive scaffolding that sustains long-term habits.

The growing interest in habit change has been accompanied by a series of conceptual and practical challenges. Niedderer et al [53] provided a macrolevel overview of behavior change design practices and noted major gaps in shared understanding, terminology, and implementation standards across sectors. This fragmentation is especially evident when comparing health-based interventions with HCI-focused strategies. For instance, while health researchers often emphasize measurable outcomes such as physical activity levels or sleep duration, HCI researchers tend to focus on experiential metrics such as emotional resonance or usability satisfaction. This inconsistency complicates the evaluation of intervention efficacy and limits the generalizability of findings.

Pinder et al [54] similarly noted the heterogeneity in how the concept of habit change is interpreted and operationalized across disciplines. In response, they proposed the “habit alternation model,” which integrates 3 dominant theories—dual-process theory, modern habit theory, and goal-setting theory—into a single explanatory framework. This model has proven influential in reconciling divergent approaches and in guiding the design of more cohesive and context-sensitive interventions.

In parallel, a growing number of behavioral scientists have emphasized the importance of integrating HCD into behavior change systems [55]. HCD techniques—particularly participatory design methods—are increasingly used to ensure that interventions align with users’ lived experiences, values, and goals. By involving users directly in the design and refinement process, these approaches seek to address the limitations of top-down, expert-driven interventions that may overlook individual motivation, digital literacy, or environmental constraints.

However, despite the growing sophistication of these approaches, the literature reveals several persistent issues. Chief among them is the challenge of sustaining user engagement over the long term and producing consistent results across diverse user populations [44,56,57]. While personalization features—such as content adaptation, feedback customization, and dynamic goal-setting—are widely used, the findings indicate that tailoring alone is insufficient. Systems that fail to account for users’ deeper cognitive and emotional processes—such as internal motivation, self-regulation, and reflective goal alignment—often achieve only short-term success before engagement tapers off.

Another persistent concern is the lack of standardization in how success is measured. Some studies report efficacies in terms of quantitative behavior change (eg, reduced screen time and increased steps per day), while others emphasize qualitative improvements (eg, enhanced focus, better mood, or increased self-awareness). This lack of convergence around evaluative criteria undermines the comparability of results and highlights the need for more unified, theory-driven frameworks for assessing intervention outcomes.

In this regard, the literature points toward the need for deeper integration of behavioral theory with longitudinal user evaluation. The most promising studies combine short-term intervention efficacy with sustained reflection and feedback loops that help users internalize new behaviors. For example, systems that facilitate reflective journaling, goal adjustment, or feedback calibration were more likely to show lasting impact. These findings underscore the value of designing for not only behavioral compliance but also behavioral understanding and transformation.

In summary, the design for behavioral changes in the context of intelligent systems represents a maturing area of research—one that is increasingly grounded in theory, enriched by participatory methods, and informed by both real-time data and long-term evaluation. While notable progress has been made in creating adaptive, user-centered systems, the next frontier will require more holistic approaches that synthesize behavioral theory, co-design practices, and robust measurement strategies to enable durable and meaningful improvements in users’ digital well-being.

#### About BIT

Third, the recent rise of BITs marks a significant and structured subdomain within the broader discourse of digital well-being, especially in health care and clinical settings. Defined by Mohr et al (2014) [58] as “the application of behavioral and psychological intervention strategies by using technological features to target behavioral, cognitive, and affective context and environment that support physical, behavioral, and mental health,” BITs have gained traction through the development of computing systems designed to support health-related behavior change. This growth has enabled behavioral scientists to generate new empirical insights on how digital tools can promote physical, emotional, and psychological well-being [36,58,59].

The review of 240 papers revealed that 55 (23%) studies explicitly focus on BIT development and evaluation, making it one of the most clinically grounded areas in the literature. These systems typically emerge from interdisciplinary collaborations among behavioral scientists, HCI researchers, and system developers, often underpinned by robust theoretical foundations. Common targets of BITs include mental health symptom reduction [60], emotional regulation, and adherence to behavioral protocols.

In recent years, BITs have evolved to incorporate technologies categorized under PITs and digital medicine. Several systems in this group exemplify the practical application of such theories through adaptive interfaces that personalize nudges, prompts, or feedback mechanisms in real time. These include systems that leverage decision science and data science to personalize interventions based on user behavior and preference data [24,61,62]. For instance, the deployment of Just-In-Time Adaptive Interventions (JITAIs)—where prompts are delivered based on biometric signals, app usage data, or behavioral cues—has become a dominant trend [63-66]. These JITAI-based systems aim to maximize user engagement by intervening at moments of high receptivity, demonstrating significant promise in both user satisfaction and short-term efficacy.

Despite their strengths, BITs continue to face several limitations. Traditionally, these technologies have been developed and evaluated within clinical contexts, emphasizing short-term efficacy over broader UX or long-term impact [67]. Researchers and practitioners have highlighted several persistent issues: (1) limited theoretical grounding in some system designs, (2) insufficient models for evaluating user interaction and engagement, (3) accessibility challenges and associated costs, (4) inconsistent user adherence, and (5) poor integration into broader health delivery infrastructures [48].

Moreover, there is a notable lack of diversity in the populations studied. Most BITs have been designed and tested in high-income settings, raising concerns about their generalizability and inclusivity. Addressing this gap, recent work has begun to explore the use of generative AI and low-cost sensing technologies to extend the reach of BITs in underserved or resource-constrained environments.

In response to these limitations, behavioral scientists have called for a shift toward more ethically grounded and human-centered BIT development. This includes integrating fragmented design perspectives into cohesive theoretical frameworks and emphasizing transparency, adaptability, and co-design practices [68]. Features such as explainable AI, user override functions, and participatory evaluation have been proposed to mitigate ethical concerns related to manipulation, autonomy, and privacy.

Synthesizing insights from BITs and broader literature on intelligent computing systems for digital well-being, 3 key observations emerge. First, much of the prior research has focused on the outcomes of behavioral decision-making without adequately considering the diversity of users. There is now increasing emphasis on individual differences—such as psychological profiles, health status, and behavioral tendencies—to inform more effective and equitable system design [53,69]. Second, while earlier work emphasized generic system usability, recent studies highlight the importance of well-structured, personalized interventions that account for contextual variables, such as environment and time of use [36,59,62,70]. Third, as AI technologies become more pervasive, researchers and practitioners are increasingly grappling with how to reconcile advancements in human autonomy research with digital well-being initiatives—particularly concerning personalized UXs and human-centered AI system design [3,67,68].

#### Finding 2. Conceptual Diversity on Human Autonomy With AI-Powered Intelligent Systems

The review results revealed that as AI-powered computing systems become increasingly integrated into our daily life—supporting decision-making, automating routine tasks, and influencing behavioral patterns—the concept of autonomy has emerged as a central design value [19,27,71]. However, the interpretation of autonomy varies widely across disciplines, leading to conceptual ambiguity and design inconsistencies. From the perspective of psychology and behavioral science, the concept of human autonomy is considered one of the basic psychological needs, particularly within the framework of self-determination theory, which views autonomy as freedom from external control or influence—including from other people and environmental factors [72]. From this perspective, psychologists and behavioral scientists have focused on behavioral interventions to change people’s attitudes and behaviors in positive ways by investigating the relationship between human autonomy and the behavioral decision-making process [47].

The analysis of 240 papers found that 147 (61%) studies engaged directly with autonomy-related issues, with 2 dominant and sometimes competing perspectives emerging: (1) technological autonomy from human control and (2) human autonomy through technology.

The first perspective, technological autonomy from human control, is most prevalent in the fields of computer science and engineering. In this framing, autonomy refers primarily to the capabilities of AI systems themselves—their ability to function independently, adapt dynamically to changing conditions, and minimize the need for continuous human input [27,73]. This notion is often associated with intelligent automation, self-regulating algorithms, and predictive systems that optimize for performance and reduce cognitive load for users. Forty-six (19%) studies aligned with this perspective, emphasizing the design of systems that learn and act autonomously, such as context-aware recommendation engines, automated productivity tools, and self-adjusting health monitors. However, while this approach offers efficiency and scalability, it also raises concerns about diminishing user control, consent, and interpretability. Systems designed for technological autonomy may inadvertently undermine user agency by prioritizing automation over transparency or flexibility—especially when users are not able to understand, override, or adapt the system’s decisions.

In contrast, the second and more human-centered perspective, human autonomy through technology, is emphasized in the disciplines of management, HCI, and HCD. This framing views technology not as a substitute for human agency but as an enabler of it—providing tools that amplify users’ capacity to make intentional, informed, and self-endorsed decisions [61,74]. In total, 102 (43%) studies in the dataset foregrounded this approach, suggesting that AI systems should support user autonomy by offering decision-support dashboards, intelligent filtering mechanisms, PITs, and customizable experiences [27,75,76]. In this view, autonomy is not merely preserved in the face of automation but is actively cultivated through interactions that respect the user’s goals, values, and contexts. Systems aligned with this perspective often use noncoercive prompts, user-configurable settings, and ethical nudging strategies to support reflection and volition rather than compliance or behavioral control [61,74]. This divergence in perspectives is summarized in Table 2.

**Table 2:** Conceptualizations of human autonomy in AI^a^ systems.

Autonomy perspective	Disciplinary origin	Papers, n	Core ideas	Key example use cases
Technological autonomy from human control	Computer science and engineering	46	AI systems operate independently, reducing user input	Predictive automation and context-aware recommender systems
Human autonomy through technology	Management, HCI^b^, and HCD^c^	102	AI enhances user capacity and supports informed and volitional action	Personal informatics, decision-support interfaces, and nudging tools

^a^AI: artificial intelligence.

^b^HCI: human-computer interaction.

^c^HCD: human-centered design.

Despite widespread efforts to embed autonomy-supportive features into intelligent systems, many researchers and practitioners continue to struggle with translating these abstract values into coherent design strategies [17,71]. The conceptual gap between the system-centered and user-centered views of autonomy contributes to ongoing tensions in system development, particularly when trying to balance the benefits of automation with the preservation of user agency.

A recurring issue highlighted in the literature is the persuasion-autonomy dilemma, where AI systems intended to assist or influence behavior (eg, through nudges or reminders) risk being perceived as overly controlling, opaque, or misaligned with users’ intentions. In practice, users often appreciate automation that reduces effort but become frustrated or resistant when systems overstep—such as enforcing goals that feel inappropriate, invasive, or difficult to override. For example, the review identified cases where health-monitoring applications encouraged physical activity without sensitivity to context (eg, illness), or algorithmic management systems in gig work environments reduced perceived autonomy and flexibility—prompting resistance among users [41,77].

These examples illustrate that perceived fairness, transparency, and reversibility often matter more to users than algorithmic sophistication alone. The review underscores that user trust and acceptance are closely linked to how much control and interpretability a system allows. In response to these concerns, a promising development is the emergence of “repairable AI”—systems that embed mechanisms for user revision, feedback, or override. These systems transform autonomy from a static feature into a relational and dynamic property, allowing users to negotiate control over time.

For instance, the TypeOut system by Xu et al [78], implemented self-affirmation prompts that allowed users to reflect on their values and motivations when trying to reduce smartphone overuse. This approach supported users’ autonomy by reinforcing self-directed action rather than imposing strict limitations. Similarly, several studies explored hybrid intelligence systems that combine algorithmic recommendations with human coaching or collaborative decision-making. These systems demonstrated higher user satisfaction, engagement, and personalization, as they allowed users to maintain interpretive authority while benefiting from intelligent assistance.

Across the dataset, the most effective systems for supporting autonomy shared several characteristics: they offered clear explanations of system behavior, allowed users to customize settings or feedback modalities, and supported decision-making rather than unilateral automation. Autonomy was thus conceptualized not as a binary variable but as a continuum that must be calibrated to the user’s needs, task context, and preferences.

In summary, the review suggests that autonomy in AI-powered systems should be approached as a relational, context-sensitive construct shaped through the dynamic interplay of system design, user interaction, and ethical affordances. While technological autonomy from human control may offer performance benefits, the long-term sustainability of human-centered AI depends on the extent to which these systems preserve and enhance human autonomy through technology. Future research and development should prioritize design practices that support transparency, reversibility, and participatory engagement, ensuring that users retain meaningful agency in an increasingly intelligent world.

#### Finding 3. Developing and Evaluating Process for Digital Well-Being With AI Systems

As AI systems become increasingly embedded in everyday life, the challenge of ensuring that these technologies genuinely support digital well-being has taken on new urgency. Existing initiatives have repeatedly highlighted a persistent gap between high-level theoretical ideals and the actionable methodologies required to guide the development and evaluation of human-centered AI systems. Two practical and recurring questions have emerged from these efforts: (1) How can researchers and practitioners identify applicable strategies for designing AI systems that enhance digital well-being? (2) How can they rigorously evaluate whether these systems are effective in achieving that goal?

Despite a growing body of literature advocating for human-centered values in AI, the systematic review of 240 papers revealed that few studies translate these values into structured, repeatable design and assessment strategies. Instead, the current landscape remains fragmented—often disconnected from both theoretical frameworks and real-world user needs. In response to this problem, this paper synthesizes prior work through the lens of the SOR model, which offers a practical and conceptual bridge between research, design, and evaluation in the context of digital well-being.

As summarized in Table 3, the SOR-based framework articulates three key considerations for guiding human-centered AI system development: (1) using contextual differences for the stimulus component, (2) accounting for individual differences in the organism component, and (3) specifying consequential differences in the response and outcome components.

**Table 3:** Methodological focus in developing AI^a^ systems for digital well-being.

SOR^b^ component	Key research concerns	Papers, n	Representative methods	Challenges identified
Contextual difference (stimulus)	Leveraging real-time sensing and situational awareness	94	Wearables and ambient sensing	Reliability of context-detection
Individual differences (organism)	How to adapt systems to personal traits and contexts	79	Profiling, personalization, and HCD^c^	Often, surface-level traits are used only
Consequential difference (response and outcome)	Long-term changes in well-being and reflection processes	140	Multimodal evaluation and journaling tools	Rarely includes holistic well-being metrics

^a^AI: artificial intelligence.

^b^SOR: stimulus-organism-response.

^c^HCD: human-centered design.

The SOR framework was well-reflected across the dataset. Ninety-four (39%) papers focused on the integration of contextual data into system design, leveraging information from wearable devices, mobile sensors, and behavioral logs to deliver personalized and context-aware interventions. These findings align with growing interest in BITs and JITAIs that respond dynamically to real-world environments and user states. Additionally, 79 (33%) papers emphasized the importance of addressing individual variability in AI systems for digital well-being. These studies adopted approaches such as adaptive personalization, participatory design, and segmentation based on psychological traits, user preferences, or digital literacy levels. Systems that integrated this type of individual profiling were consistently associated with higher levels of user engagement, perceived relevance, and intervention efficacy. However, the third design consideration—specifying consequential outcomes—remains underdeveloped across the literature. While 140 (58%) papers discussed the interaction between context, individual traits, and well-being outcomes, many stopped short of evaluating long-term psychological or behavioral change. Instead, outcome assessments often relied on narrow usability metrics (eg, user satisfaction or screen time reduction) or short-term behavioral proxies (eg, frequency of feature use), without considering deeper indicators such as habit formation, digital resilience, or emotional regulation.

**Figure 4:**
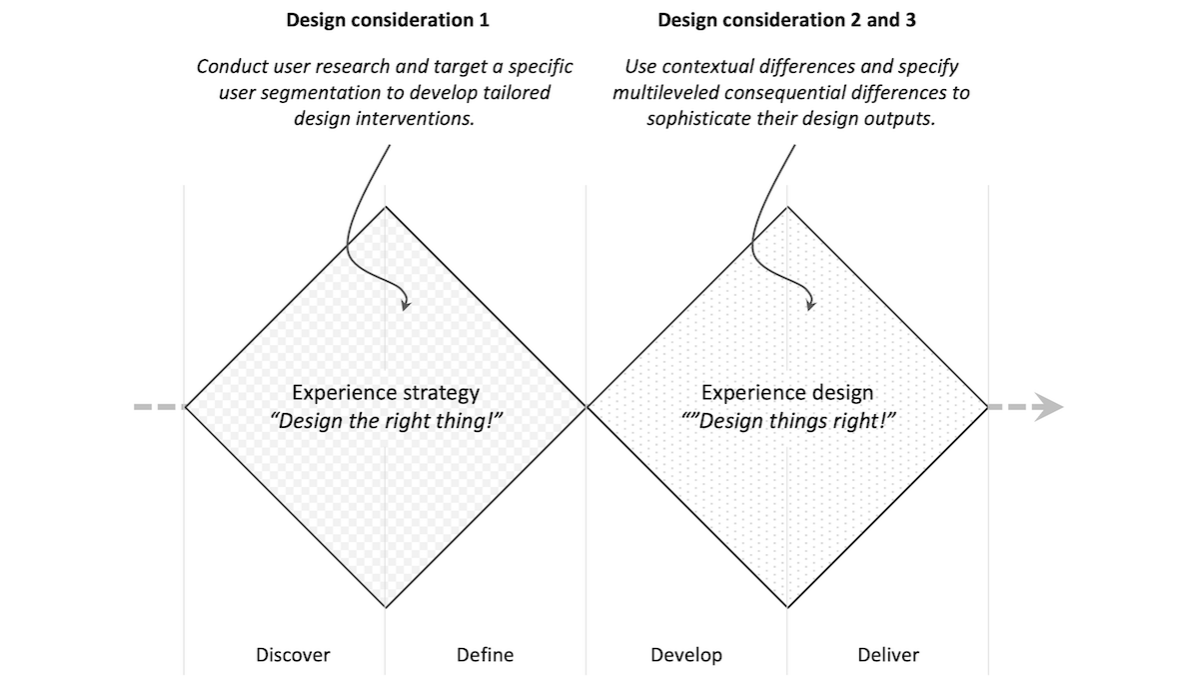
The developing process for digital well-being with AI systems. AI: artificial intelligence.

Building on these insights, the development of digital well-being interventions through AI can be conceptualized in two phases, aligned with the revamped double diamond design model [79]: (1) experience strategy—designing the right thing, and (2) experience design—designing things right (Figure 4). In the experience strategy phase, researchers and practitioners conduct user research to understand core challenges and motivations. Here, individual differences (SOR: organism) are identified through profiling and segmentation—considering factors such as personality, digital habits, emotional needs, and cognitive preferences. These insights help define targeted user groups and establish design priorities.

The experience design phase focuses on implementation—developing system features that respond to both individual and contextual variation (SOR: stimulus). This includes creating adaptive interfaces, intelligent feedback loops, and multimodal interventions that nudge users toward meaningful behaviors. As part of this phase, teams are encouraged to define consequential outcomes (SOR: response and outcome) not just in terms of app usage or time-on-task, but through broader and more enduring metrics such as user autonomy, emotional well-being, and sustainable habit change.

Despite the clear utility of this model, the review found that many studies continued to rely on simplistic evaluation strategies, such as postuse surveys or single-time-point measurements. While these can offer useful feedback, they often fail to capture the temporal and emotional dynamics that characterize digital well-being. For instance, AI systems intended to reduce screen time were rarely evaluated for their effects on users’ perceived autonomy or cognitive fatigue. Likewise, productivity tools often neglect to measure whether increased efficiency was accompanied by psychological strain or decision exhaustion.

Additionally, promising developments were observed in systems that integrate generative AI for personalized support in mindfulness, reflection, and journaling tasks. These tools showed early evidence of enhancing emotional regulation and user engagement. However, consistent frameworks for evaluating their effects—particularly on user autonomy and mental energy—remain lacking. Furthermore, despite increased technical sophistication, emotion recognition algorithms still struggle with reliability, and few studies incorporated robust longitudinal evaluations. Equity concerns also surfaced across the dataset. Very few evaluation frameworks accounted for variables such as socioeconomic status, neurodiversity, or differential access to digital tools, raising concerns that certain AI interventions may inadvertently reinforce existing disparities rather than reduce them.

In conclusion, the development and evaluation of AI systems for digital well-being must move beyond high-level rhetoric and toward deeply contextualized, empirically grounded processes. By applying frameworks such as the SOR model and drawing from findings in interdisciplinary literature, researchers and practitioners can more effectively align design goals with meaningful, measurable, and user-centered outcomes. The field’s advancement depends not only on technical innovation but also on a more nuanced understanding of how intelligent systems intersect with users’ daily decisions, emotional states, and broader societal contexts.

### Development of HCAI-DW Model

#### Background

The preceding 3 findings collectively underscore a fragmented but evolving landscape in the design and evaluation of intelligent systems for digital well-being. First, implementation efforts tend to cluster around 3 key domains—supporting well-being in daily HCIs, designing for behavioral and habit change, and deploying BITs—but each varies in conceptual clarity, theoretical grounding, and methodological rigor. Second, the value of user autonomy is widely recognized, but interpreted through different disciplinary lenses, leading to tensions between automation and agency. Third, despite strong calls for human-centered development, the field still lacks coherent, actionable methodologies for designing and assessing AI systems that meaningfully support users’ emotional, behavioral, and cognitive well-being.

In response to these interrelated challenges, this study proposes an integrative framework: the HCAI-DW model. Developed to bridge the persistent gaps between conceptual ideals and real-world implementation, the HCAI-DW model synthesizes insights from empirical findings, behavioral theory, and interdisciplinary practice. It offers a structured lens to guide researchers and practitioners in designing, deploying, and evaluating AI-powered systems that promote human flourishing in digital environments.

The model was developed through an iterative, participatory process that combined the systematic review of 240 research papers with expert feedback from 5 professors and 17 graduate students in HCI and HCD. These structured review sessions—conducted at both early and final stages of the modeling process—played a critical role in refining the model’s conceptual clarity, usability, and relevance to current challenges. The goal was not only to reflect the state of the field but to cocreate a framework that is both theoretically robust and practically applicable.

A key insight that emerged throughout this process is that digital well-being is inherently multidimensional, shaped by the complex interplay of individual psychological traits, system-level design interventions, users’ behavioral responses, and broader social or environmental outcomes. To capture this interplay, the HCAI-DW model adapts and extends the SOR model, a well-established paradigm in behavioral science, and reframes it for the context of human-AI interaction. This adaptation provides a structured but flexible blueprint for understanding how intelligent systems can shape and support digital well-being in everyday life.

#### Overview

**Figure 5:**
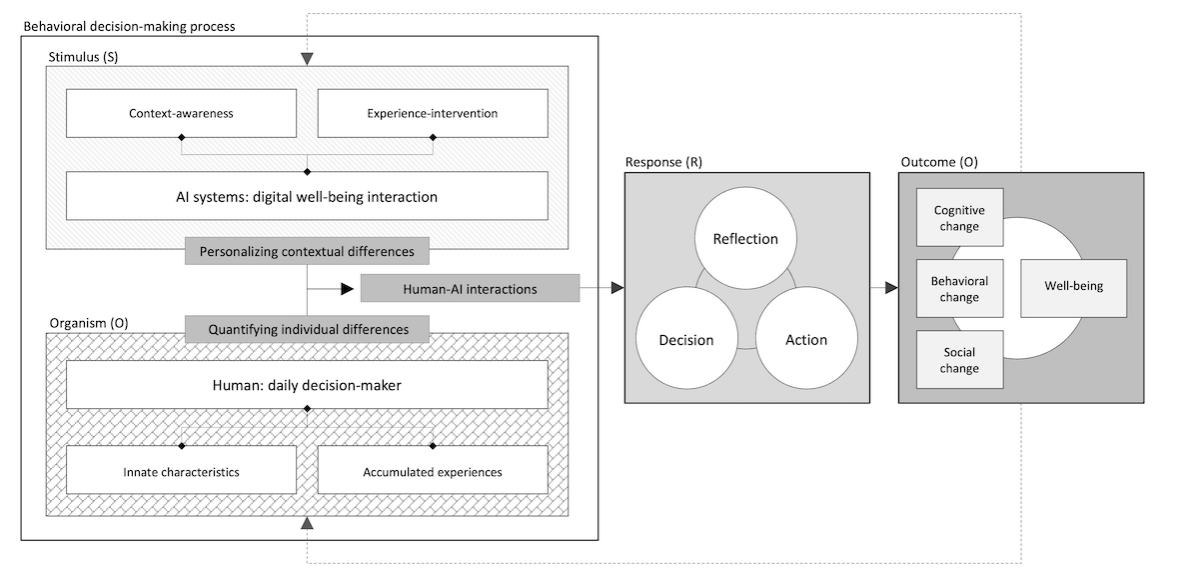
HCAI-DW Model. AI: artificial intelligence; HCAI-DW: human-centered artificial intelligence for digital well-being.

As illustrated in Figure 5, the HCAI-DW model describes 4 essential and interrelated components that shape human-centered AI system development for digital well-being: (1) the organism component, which views users as daily behavioral decision makers; (2) the stimulus component, which frames AI system features as intentional behavioral interventions; (3) the response component, which introduces a cyclical model of user engagement involving decision, action, and reflection; and (4) the outcome component, which emphasizes a layered understanding of cognitive, behavioral, and social transformations. Together, these components offer a comprehensive pathway for designing intelligent systems that promote digital well-being in nuanced, personalized, and sustainable ways.

#### Organism: Daily Decision Makers

The organism component foregrounds the diversity of individual characteristics that influence how people interact with AI-powered systems. In the HCAI-DW model, these differences—including traits such as motivation, digital literacy, emotional state, and decision-making tendencies—are recognized as critical design inputs for tailoring interactions. As past research has demonstrated, individuals tend to make behavioral decisions based on their innate characteristics or accumulated experiences. Profiling user preferences and behaviors allows AI systems to deliver personalized support that is more effective and meaningful in fostering digital well-being [36,59]. The reviewed literature shows that most existing studies emphasized the importance of personalization, though only a fraction deployed adaptive systems that responded to individual differences in real time. By leveraging machine learning and deep learning algorithms, intelligent systems can move beyond generalized models to provide highly contextualized and evolving interventions [59,80,81]. This model suggests treating AI systems not merely as tools for behavior modification but as coregulatory agents that continuously adapt to users as they navigate complex decision-making environments. For this reason, the HCAI-DW model posits that seeing users as “daily behavioral decision makers” is essential for designing effective AI systems that address the nuances of digital well-being.

#### Stimulus: Digital Well-Being Interactions

The stimulus component redefines AI system features as intentional behavioral interventions that can promote or hinder digital well-being. While many previous initiatives have explored user interaction with AI as a design theme or an evaluation component [3,82], the HCAI-DW model differentiates between 2 interrelated stimulus phases: the context-awareness phase and the experience-intervention phase.

The context-awareness phase is derived from the quantified-self and personal informatics movements [59]. In this phase, AI systems track and interpret user data—including time, location, physical state, and social context—to deliver interventions that are sensitive to situational dynamics. Of the 240 reviewed studies, 94 specifically focused on the integration of such contextual variables into design. Wearable sensors, app usage logs, and mood-tracking tools were among the techniques used to improve the precision of interventions. The experience-intervention phase draws from BIT literature [70,83]. In this phase, AI systems deliver tailored and systematized interactions that aim to encourage behavioral shifts and preserve user autonomy. These interventions include nudges, reminders, content recommendations, and interface adjustments. Rather than enforcing a one-size-fits-all approach, the HCAI-DW model supports dynamic and transparent adaptation to individual feedback, reducing the risk of perceived manipulation or coercion.

Together, these 2 stimulus phases highlight the dual nature of AI system design: sensing and acting. Systems that only track without intervening, or intervene without contextual sensitivity, risk ineffectiveness or user resistance. The HCAI-DW model provides a roadmap for designing stimulus mechanisms that are integrated, adaptive, and user-centric.

#### Response: Decision-Action-Reflection Loop

The response component expands the understanding of user engagement beyond simplistic stimulus-response paradigms to include a full cycle of decision, action, and reflection. This loop is crucial for supporting sustainable behavior change and deeper cognitive engagement. Most previous studies have treated user responses as fixed behavioral outputs (eg, goal achievement and app usage). However, this approach overlooks the underlying reflective processes that support long-term well-being [16,36]. In contrast, the HCAI-DW model emphasizes the temporal and cyclical nature of engagement. AI systems should facilitate not only behavioral outcomes but also opportunities for users to reflect on their choices, understand system recommendations, and adjust their goals over time [17,19,53]. Such features are underdeveloped in the current literature; only few studies described systems that supported ongoing reflective practice, and even fewer implemented adaptive feedback based on longitudinal user behavior. Examples of effective reflective design include journaling prompts, feedback dashboards, and generative content that encourages users to process their experiences emotionally and cognitively. These elements help close the loop between system recommendation and user transformation, offering pathways for deeper and more enduring engagement.

#### Outcome: From Cognitive Change to Social Change

The outcome component identifies and categorizes the types of changes users experience after interacting with AI-powered systems [4,71]. Based on an analysis of prior research, the HCAI-DW model distinguishes 3 levels of outcome: cognitive change (eg, increased awareness and motivation), behavioral change (eg, reduced screen time and improved sleep), and social change (eg, enhanced family communication and digital equity). While many previous approaches target a single optimized outcome (eg, productivity or screen-time reduction), the HCAI-DW model encourages consideration of multilayered and user-defined metrics. It also addresses a common limitation of models such as Motivation, Engagement, and Thriving in User Experience model [27], which, despite offering a comprehensive structure for evaluating technology-mediated experience, struggle with implementation due to insufficient differentiation between experience levels and limited guidance on design implications.

This model fills that gap by suggesting a framework for systematically specifying consequential differences—that is, the variations in outcomes that result from users’ unique behavioral decisions and reflective processes. Many prior interventions have focused on training or instructing users to modify their behavior [4,35] but have not always aligned these goals with users’ lived experiences, values, or autonomy. The HCAI-DW model enables researchers and practitioners to avoid these pitfalls by distinguishing between process (decision-making) and product (outcome) and ensuring that both are addressed in a human-centered, contextualized manner.

In sum, the HCAI-DW model offers a synthesized and actionable framework that integrates the diverse findings of this review and provides a comprehensive guide for the design, development, and evaluation of intelligent systems that prioritize digital well-being. Its structure—rooted in the SOR model and refined through interdisciplinary and participatory input—reflects a growing need in the field for models that are not only conceptually rigorous but also practically useful. By highlighting individual differences, incorporating contextual adaptation, supporting reflective engagement, and mapping layered outcomes, the model promotes a vision of AI that respects human agency while optimizing support. As AI technologies become increasingly embedded in everyday life, models such as HCAI-DW will be critical in guiding their ethical, effective, and human-centered development.

## Discussion

### Principal Findings

This paper developed the HCAI-DW model to conceptualize how AI systems can support individuals’ behavioral decision-making and foster behavioral changes that enhance their daily digital well-being. Grounded in evidence-based academic literature and established perspectives from relevant practical domains, this model reflects a human-centered approach to AI systems aimed at improving digital well-being. The HCAI-DW model is expected to enhance existing technology development practices by offering a systematic framework to encode and use core factors that drive digital well-being in AI computing systems. Furthermore, the model was purposefully designed for intuitive and practical use, addressing the emerging needs of researchers and practitioners seeking to apply theoretical insights and design approaches to shape human experiences. As such, the model enables the creation of more effective AI system interactions by tailoring them to individual and contextual differences, particularly those associated with decision-making processes.

### Limitations

This paper offers a timely investigation into human-centered AI systems designed to support users’ digital well-being, incorporating insights from a systematic literature review. To provide a comprehensive and multidisciplinary overview of the most relevant research, 4 academic databases were used as the primary sources for data collection. This approach aimed to ensure the inclusion of diverse scholarly contributions from computing, design, behavioral science, and information systems. However, a key limitation lies in the fragmented nature of existing studies in this domain. Many initiatives related to digital well-being and intelligent computing systems are published under varied terminologies and conceptual framings. To address this, the initial phase of data collection used broadly defined search terms to capture a wide scope of literature. Despite this inclusive strategy, there is a possibility that some relevant studies—particularly those published under domain-specific or alternative terminologies—were not included. For example, literature from niche fields or interdisciplinary collaborations may have described similar phenomena without explicitly using the terms “digital well-being” or “AI systems.”

Additionally, while the topic has gained increasing attention in both academic and industrial contexts, the number of studies explicitly integrating the concepts of AI and digital well-being remains relatively limited. This restricts the ability to generalize findings across all potential applications or use cases of AI systems designed for human well-being. Future systematic reviews should aim to capture these additional perspectives by refining search protocols and expanding the inclusion criteria to account for heterogeneity in terminologies, research methods, design strategies, usage contexts, and evaluative outcomes.

Furthermore, the HCAI-DW model proposed in this study should be understood as a preliminary step rather than a finalized theoretical framework. The model synthesizes key themes and design considerations from the existing body of work to propose an integrated perspective on designing for digital well-being with AI. However, the model has not yet been empirically validated, and its applicability across different cultural, technological, or organizational contexts remains uncertain. This limitation suggests that further empirical research—particularly studies conducted in real-world design and development settings—is necessary to test and refine the model’s relevance, accuracy, and usability.

In summary, while this paper contributes a foundational understanding of how human-centered AI systems can support digital well-being, the evolving and interdisciplinary nature of the field calls for continued theoretical expansion and empirical validation. Follow-up works should also explore additional stakeholder perspectives and context-specific requirements to enhance the robustness and applicability of the HCAI-DW framework.

### Future Directions

#### Overview

This study provides a foundation for advancing both theoretical and practical efforts in designing AI systems that promote digital well-being in daily HCIs. Based on insights from the systematic literature review, three key challenges emerge for future research and practice: (1) developing new evaluation methods for digital well-being in AI contexts, (2) addressing ethical considerations in AI-powered technology interventions, and (3) designing for hyper-personalization by accounting for individual decision-making contexts. The following sections elaborate on each of these challenges and outline opportunities to enrich the body of knowledge in this domain.

#### New Evaluation Methods for Digital Well-Being in AI Systems

Several studies have highlighted the limitations of existing evaluation tools for measuring subjective experiences of well-being in technology use. In particular, concerns have been raised regarding the reliability and validity of commonly used instruments [35,83]. From the perspective of the HCAI-DW model, digital well-being outcomes are shaped by a complex interplay of daily behavioral decisions and interactions with AI systems. However, a major challenge remains: there is a lack of well-validated and universally applicable measurement tools that can accurately assess the short-term and long-term effects of AI-based interventions.

Current tools are often adapted from adjacent domains and may not reflect the nuances of digital well-being in AI-enhanced contexts. As a result, the evaluation of AI system effectiveness remains inconsistent and difficult to standardize [84]. There is an urgent need to develop novel, context-sensitive evaluation methods that can capture the dynamic and individualized nature of well-being outcomes. These tools should account for both objective indicators and subjective experiences to ensure that the impact of AI systems on users’ digital well-being can be meaningfully assessed.

#### Ethical Considerations in AI-Powered Interventions

While digital well-being aims to enhance users’ lives through technology, intervention through AI systems must be approached with caution. Interventions, though potentially beneficial, are inherently value-neutral and may be used in manipulative or unethical ways [85]. For example, leveraging behavioral decision-making mechanisms could enable governments or corporations to influence individuals’ choices in ways that serve organizational goals rather than personal well-being [54]. This raises significant ethical concerns, including the risk of undermining users’ autonomy or reinforcing harmful behavioral patterns [86].

The HCAI-DW model calls for AI systems that are sensitive to individual needs and contexts. However, achieving this level of personalization often requires collecting sensitive behavioral data, which brings digital privacy into focus. Ethical design practices must address questions of informed consent, data transparency, and user agency. Researchers and practitioners should ensure that AI interventions prioritize individual rights and safeguard against potential misuse or harm. Privacy-by-design principles and responsible data governance should be core components of future development strategies.

#### Designing for Hyper-Personalization Based on Individual Decision-Making Contexts

The advent of AI has fueled a growing trend toward hyper-personalization—tailoring content, services, and experiences to individual users based on real-time data and behavioral histories [17,18,62]. Although hyper-personalization holds promise for promoting sustained engagement and behavioral change, few studies have explored its implementation through the lens of digital well-being and human-centered AI [17,53,74].

The HCAI-DW model offers a conceptual foundation for understanding how personalization can support well-being by considering users’ individual, contextual, and behavioral differences. It emphasizes that design should not only respond to users’ past behaviors but also anticipate their goals, motivations, and environmental conditions. To fully realize the potential of hyper-personalization in AI systems, future research should identify actionable design principles that align AI-driven personalization with users’ well-being outcomes. This includes developing frameworks for adaptive and context-aware personalization that respect user boundaries and preferences. By doing so, designers can increase the impact of AI systems on sustained behavioral change and help users make informed decisions that enhance their overall digital well-being.

### Conclusions

This paper identifies everyday behavioral decision-making as a foundational concept for designing AI systems that enhance digital well-being. By synthesizing fragmented and often inconsistent perspectives from prior research, the study introduces the HCAI-DW model. This model offers a conceptual framework that links individual behaviors, AI system interactions, and well-being outcomes in everyday life. Through this model, this paper demonstrates how AI systems can be intentionally designed to support users’ behavioral decisions in ways that promote meaningful and sustained improvements in digital well-being. Rather than treating digital well-being as an abstract or isolated construct, the HCAI-DW model emphasizes its dynamic and context-dependent nature, highlighting the critical role of personalized, ethical, and adaptive AI design. Ultimately, this work encourages researchers and practitioners to reconsider the evolving relationship between humans and AI in everyday contexts. It invites further exploration into what it means to be a “new type of human”—one whose daily life and well-being are increasingly mediated by intelligent technologies.

## Appendix

Multimedia Appendix 1 The list of the reviewed 240 articles in the study.

Multimedia Appendix 2 PRISMA checklist.

## Abbreviations

ACM: Association for Computing Machinery
AI: artificial intelligence
BIT: behavioral intervention technology
HCAI-DW: human-centered artificial intelligence for digital well-being
HCD: human-centered design
HCI: human-computer interaction
ICT: information and communication technology
JITAI: Just-In-Time Adaptive Intervention
PIT: personal informatics tool
PRISMA: Preferred Reporting Items for Systematic Reviews and Meta-Analyses
SIGCHI: Special Interest Group on Computer-Human Interaction
SOR: stimulus-organism-response
UX: user experience
